# Exploration of techniques for the enhancement of latent fingermarks from fired and unfired cartridge cases: a systematic review

**DOI:** 10.1093/fsr/owaf006

**Published:** 2025-02-17

**Authors:** Maxwell Abedi, Christopher Mabasa, Sekgololo A Mabudusha

**Affiliations:** Department of Forensic Sciences, University of Cape Coast, Cape Coast, Ghana; Department of Police Practice, University of South Africa, Pretoria, South Africa; Department of Police Practice, University of South Africa, Pretoria, South Africa; Department of Police Practice, University of South Africa, Pretoria, South Africa

**Keywords:** forensic sciences, fingermark recovery, fingermark development, fingermark enhancement, ballistics, fired cartridge case, unfired cartridge, firearm, latent fingermark

## Abstract

The ability to develop latent fingermarks from fired and unfired cartridge cases can be crucial in resolving crime cases and advancing forensic investigations. Currently, there is a lack of consensus on the ideal technique to employ for the enhancement of latent fingermarks from fired and unfired cartridge cases. This review therefore aims to explore techniques and methods employed to develop latent fingermarks from fired and unfired cartridge cases. A systematic search of peer-reviewed original articles was performed from four main electronic databases: ScienceDirect, Scopus, Google Scholar, and PubMed. According to data from our review, the most well-established method for developing latent fingermarks from fired and unfired cartridge cases remains the sequential application of cyanoacrylate fuming, followed by gun bluing, and the application of a fluorescent dye called basic yellow 40. This review also discusses the current scope of research, highlights the limitations, and provides practical recommendations for future perspectives.

**Key points**
 

- Fingermark evidence on fired and unfired cartridge cases cannot be undervalued.
- The enhancement of latent fingermarks from fired cartridge cases is possible although challenging.
- Enhancement of latent fingermarks from fired cartridge case is possible with cyanoacrylate fuming followed by gun bluing and basic yellow 40.
- Recover Latent Fingerprint Technology, palladium deposition, and cold patination fluid are promising fingermark enhancement techniques.

## Introduction

Gun crimes such as homicide, suicide, accidental gun firing, armed robbery, and often mass shootings have been a persistent and chief cause of mortality across all age groups globally [1, 2]. For example, Cunningham et al. [3] reported that ⁓18 000 people aged ≤17 years in the US die annually from firearm-related crime (mainly from accidental gun firing). Further, a retrospective study spanning from 2004 to 2014 in South Africa (KwaZulu-Natal) by Shamase et al. [4] disclosed that ⁓19% representing 4 087 out of 22 910 of all deaths was ascribed to firearm-related injuries. In Ghana, only 1.7% (*n* = 90) of firearm-related fatalities were recorded following 5 359 autopsies conducted between 2008 and 2013 [5]. The upsurge in firearm-related crime globally has been linked to the ease of access to firearms and ammunition due to weak regulations and the lack of operational policy on firearm and ammunition possession [1] coupled with the advent of 3D printed guns [6].

The preponderance of physical evidence recovered from such firearm-related scenes comprises fired cartridge cases, unfired cartridges, fired projectiles (bullets), and often firearms [7]. The ballistic investigation of the foregoing evidence primarily involves linking the fired cartridge case or the fired bullet to the suspected firearm [8]. However, forensic identification of the shooter or persons who have come into contact with the firearm and its components goes beyond just the firearm-ammunition linkage [9]. During crimes involving firearms, it is not uncommon for perpetrators to leave behind their fingermarks on the firearm and ammunition (mainly the casing). Although criminals have employed precautionary acts [10], such as wearing gloves and cleaning the firearm post-use to destroy forensic evidence, the ammunition is presumably loaded into the chamber or magazine of the firearm with the bare hands [11, 12], consequently leaving behind latent fingermarks on the ammunition and parts of the firearm.

The ability to develop latent fingermarks on fired and unfired cartridge cases can be crucial in resolving crime cases and advancing forensic investigations. Despite the success in a few forensic cases [7, 13–15], the development of identifiable fingermarks with continuous ridges on fired cartridge cases remains challenging. Previous studies [7, 15–17] have ascribed this drawback to several factors. For instance, Girelli et al. [7] indicated that the handling characteristics of the ammunition by the shooter recurrently result in the deposition of smudged or smeared and sometimes imbricated partial fingermarks which may be subsequently deteriorated during the firing process. Furthermore, the frictional contact between the ammunition and walls of the firearm chamber plus the ejection mechanism causes the worst damage to the fingermark on the fired cartridge cases [7, 16].

Over the past decade, literature [7, 14, 15] has emerged that explores the potential of different fingermark enhancement techniques for the development of latent fingermarks on fired and unfired cartridge cases. Notably, Dominick and Laing [14] showed that “cyanoacrylate fuming (CA) followed by gun bluing (GB), and basic yellow 40 (BY40)” and “CA followed by palladium deposition (PD)” are efficient techniques for the enhancement of latent fingermarks from unfired cartridges. This was consistently supported by the works of Girelli et al. [7, 15]. Further, many standalone fingermark enhancement techniques such as vacuum metal deposition (VMD) [18], electrodeposition of poly(3,4-ethylene dioxythiophene) [19], electrostatic adsorption technique [20], cold patination fluid (CPF) [21], and RECOVER Latent Fingerprint Technology (RLFT) [22] have produced promising results.

The merit notwithstanding, there is a current lack of consensus on the ideal technique to adopt for the recovery of latent fingermarks on fired and unfired cartridge cases. This review therefore aims to compile empirical data on the myriad fingermark enhancement techniques employed for developing latent fingermarks on fired and unfired cartridge cases. It also explores the efficiency of the techniques and their limitations and provides recommendations for future perspectives. The information collated is expected to aid operational forensic laboratories in making informed decisions on the most reliable fingermark enhancement technique to use for developing latent fingermarks on fired and unfired cartridge cases.

## Methods

### Registration and search strategy

This review was not registered with the International Prospective Register of Systematic Review (PROSPERO) because the study had no health-related outcome. The review was guided by the research question “What are the techniques used for developing latent fingermarks from fired and unfired cartridge cases?”. We adopted the PICO acronym (P: Population, I: Intervention, C: Comparison, and O: Outcome) to guide the development of the research question and the formulation of our search terms. The search for relevant articles was executed using Boolean operators such as “AND” and “OR” to capture a wide array of results and to ensure that primary research papers relevant to the present topic were not overlooked.

### Literature search

A highly sensitive search strategy (Table 1) was developed to obtain articles exploring techniques for enhancing latent fingermarks from fired and unfired cartridge cases. We performed a systematic search (between 5 June and 28 July 2023) of peer-reviewed original articles from four main electronic databases: ScienceDirect, Scopus, Google Scholar, and PubMed, and included papers that were published from October 1997 to December 2023. The search dates precede the actual publication dates of some referenced articles because the databases may have indexed the articles before they were officially released, and also to incorporate the latest technological literature on the subject. These databases were searched using a vast search term broadly categorised as “latent fingermark”, “fired cartridge case”, “unfired cartridge”, “fingermark recovery”, “fingermark development techniques”, “fingermark enhancement techniques”, and “fingermark development”. The papers obtained were imported into the Rayyan web tool (https://www.rayyan.ai/) for further evaluation.

**Table 1:** Search strategy employed for the formulation of search terms to search the electronic databases: ScienceDirect, Scopus, Google Scholar, and PubMed.

**Key terms**	**Expanded search items**
Latent fingermark	(“Invisible print” OR “latent fingerprint” OR “invisible fingerprint”) AND
Fired cartridge case	(“spent cartridge case” OR “casing” OR “shell casing”) AND
Unfired cartridge	(“Ammunition” OR “cartridge” OR “Round” OR “live round”) AND
Techniques/Methods	(“Fingermark detection techniques” OR “fingermark development techniques” OR “fingermark development methods” OR “fingermark recovery techniques” OR “fingermark enhancement techniques” OR “fingermark visualisation techniques”) AND
Fingermark development	“Fingermark enhancement” OR “fingermark recovery” OR “fingermark visualisation” OR “fingerprint detection” OR “fingerprint development” OR “fingerprint enhancement”

### Screening and eligibility criteria

An independent screening approach was used to facilitate the review of the results obtained from the database search. The titles and abstracts of each article were first screened by two independent reviewers (MA and CM) using the Rayyan web tool to ascertain their relevance for the present study. The articles were grouped into three namely “included”, “excluded”, and “uncertain” categories. The abstract and method section of articles in the uncertain category were perused and regrouped as either included or excluded. All original full-text articles plus selected technical notes and case reports that explored techniques for fingermark enhancement on ballistic material (fired cartridge case, unfired cartridge, firearm, and brass metal plate) were included in the study. We excluded review papers, conference papers, discussion papers, editorials, and non-research letters from the study.

## Results and discussion

### Characteristics of studies identified

**Figure 1:**
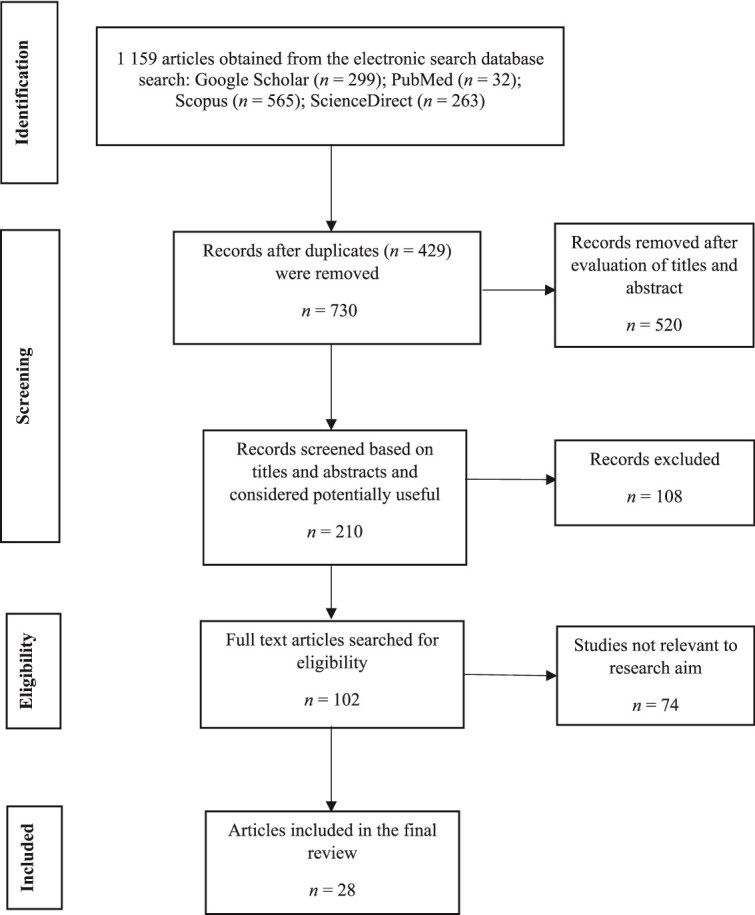
A PRISMA flow chart of the selection of studies that investigated techniques for enhancing latent fingermarks from fired and unfired cartridge cases.

Figure 1 shows the PRISMA flowchart for the study selection and screening. Overall, 1 159 studies were obtained from searching four electronic databases (Google Scholar = 299; ScienceDirect = 263; PubMed = 32; Scopus = 565). Exactly 730 unique citations remained following the removal of duplicates (*n* = 429). After critical evaluation of the titles and abstracts, precisely 210 articles were considered potentially useful for the present systematic review. Out of this, 108 articles were excluded from the study because they did not have any relevance to the study. The full text of the remaining 102 articles was further assessed and this resulted in the final removal of 74 articles (repeated in the database = 11; non-original study = 8; not related to fingermark enhancement from fired and unfired cartridge cases = 46; full articles not accessible = 9). We therefore included 28 full-text articles published in six journals that met the inclusion criteria. The majority of the studies (*n* = 9, 32.1%) were each published in the *Journal of Forensic Identification* and *Forensic Science International*, followed by the *Journal of Forensic Science* (*n* = 6, 21.4%), *Science and Justice* (*n* = 2, 7.1%), *Identification Canada* (*n* = 1, 3.6%), and *Wiley Nano Select* (*n* = 1, 3.6%). A greater number of the studies (89.3%) were published in the last decade.

### Techniques for latent fingermark enhancement on fired and unfired cartridge cases

Table 2 provides an overview of studies investigating techniques for enhancing latent fingermarks from fired and unfired cartridge cases. Fired and unfired cartridge cases present challenges that preclude the recovery and enhancement of latent fingermarks. Efforts to explore techniques ideal for enhancing latent fingermarks from fired and unfired cartridge cases date back to the 19th century. A notable example was exhibited in a laboratory study conducted by Migron et al. [17] to develop latent fingermarks on the different caliber of cartridge cases *viz* 5.56 mm, 9 mm, and 7.62 mm fired from M16, Parabellum, and AK-47 (Automatic Kalashnikov), respectively. Eccrine and sebaceous fingermarks were deposited on the cartridge cases prior to the firing process and groups of the fired cases were subsequently visualised using techniques such as soot application (powder method), PD, silver deposition, and selenation. Migron et al. [17] disclosed that the metal deposition technique (using palladium or silver) revealed identifiable sebaceous fingermarks following further visualisation with an alternative light source (ALS). The soot application (powder method) and the PD yielded zero results when attempted on cartridge cases bearing eccrine latent fingermarks. Thus, the enhancement of sebaceous fingermarks produced good-quality ridges compared to the enhancement of eccrine fingermarks. This variation in enhancement can be attributed to the fact that eccrine constituents (mainly composed of sweat) are more prone to evaporation than sebaceous constituents, hence limiting the sensitivity of the fingermark enhancement technique. Cantu et al. [23] used acidified hydrogen peroxide (AHP) comprising a mixture of weak acetic acid and hydrogen peroxide to successfully develop and visualise latent fingermarks. This outcome was supported by the research performed by Swofford et al. [28] to evaluate the impact of AHP on the enhancement of latent fingermarks from fired cartridge cases. The success notwithstanding, the authors recommended that AHP application should only be considered following the CA technique and the application of BY40 or staining with rhodamine 6G. This recommendation is in the right direction because the AHP technique is destructive and corrosive with the potential of altering the presence of vital toolmarks (chamber marks, lip marks, ejector marks, and breech face marks) on the fired cartridge case. The AHP is most sensitive to developing eccrine fingermarks through an oxidizing or etching process. Consequently, the enhancement of sebaceous latent fingermarks on the fired cases with AHP is challenging because the sebum or oil constituent resists etching, a key mechanism required for the enhancement of eccrine fingermarks [23]. It must be emphasised that the blowback of hot gases during the firing process destroys the fingermark constituent and reduces its persistence on the fired cartridge case, consequentially, affecting the sensitivity and efficiency of the selected fingermark enhancement technique.

**Table 2:** Summary of studies that explored techniques for recovering latent fingermarks from fired and unfired cartridge cases with dinstinct markers or labels used to differentiate findings.

**Studies**	**Year of publication**	**Enhancement technique**	**Results/conclusion**	**Types of fingermarks**	**Firearm/ammunition/substrates type**
Migron et al. [17]	1998	Palladium dichloride and dipotassium hexachloropalladate, magnetic powder, GB	The careful use of illumination after metal vapor deposition enabled visualisation. The powders used are inadequate for developing latent fingermarks.	Eccrine and sebaceous fingermark	M16 (5.56 mm caliber), AK-47 (7.62 × 39 mm), 9 mm Parabellum
Cantu et al. [23]	1998	AHP	AHP formulas successfully developed latent fingermarks although etched metal cartridge cases in areas with no sebaceous latent fingermark material. Application of GB to the AHP-treated cartridge case facilitated fingermark enhancement.	Not specified	Not specified
Bond and Heidel [13]	2009	Heating + potential of 2.5 kV + conducting carbon powder	Enhanced fingermark from the 9 mm casing despite the time elapsed. The technique can potentially enhance latent fingermarks on old and new cartridge cases exposed to harsh environmental conditions.	Natural fingermark	9 mm brass shell casings
Dominick and Laing [14]	2011	CA followed by BY40, CA followed by GB followed by BY40, GB only, CA followed by PD, PD only, and powder suspension	CA + GB + BY40 and CA followed by PD were the most efficient at developing latent fingermarks.	Sebaceous fingermark	0.22 cal, 0.32 cal, 9 mm, 0.38 cal, 12-gauge ribbed shotgun, and 12-gauge smooth shotgun
Bhaloo et al. [24]	2011	Electrostatic deposition technique	CA + GB + BY40 were successful at developing fingermarks on cartridge cases, with greater success observed when used on unfired cartridge cases. The enhancement efficiency of GB and PD was better than CA + BY40. The electrostatic deposition technique failed to give any results using the setup described.	Natural fingermark	Fired cartridge cases
Paine et al. [25]	2011	CA	Relative humidity of ⁓80% is essential for the development of latent fingermarks on various non-porous substrates.	Natural, sebaceous, and eccrine fingermark	Brass plate
Bond [26]	2011	Digital colour mapping	Enhanced good contrast fingermark images with the best results obtained when the brass case was heated to 250°C.	Natural fingermark	9 mm pistol/cartridge
Nizam et al. [27]	2012	Electrolysis	Enhancement with electrolysis was time-sensitive and clarity of fingermark was obtained with lesser electrolysis duration and increased acid concentration.	Not specified	Not specified
Swofford et al. [28]	2013	AHP	Obtained satisfactory results with AHP and further alluded that its application should be initiated following CA + BY40 or rhodamine 6G.	Natural fingermark	Not specified
Leintz and Bond [29]	2013	RUVIS	RUVIS was unsuccessful at developing fingermarks from fired brass cartridge cases. Optical interference and digital colour mapping were successful at visualising fingermarks from 3/12 fired brass cartridge cases.	Natural fingermark	Not specified
Girelli et al. [15]	2015	Powdering (regular powder dusting and magnetic powder application), CA, BY40, GB, and AHP solutions	Sequential application of CA, GB, and BY40 produced the best result on both fired and unfired cartridge cases. The clarity of enhanced fingermarks was better on unfired cartridge cases compared to fired cartridge cases.	Natural fingermark	Glock 17 pistol/9 mm casing and brass disc
Amata et al. [30]	2015	CA + UV light	Obtained identifiable fingermarks from firearm trigger using CA + UV light.	Natural fingermark	Mauser Werke 90 DA (9 mm Parabellum)
James and Altamimi [21]	2015	CPF	Obtained more identifiable fingermarks on live ammunition than on the fired cartridge case. Enhancement of sebaceous fingermarks was relatively more successful than those of the eccrine fingermark.	Sebaceous and eccrine fingermark	SIG Sauer P226 Pistol/ 9 mm Luger rounds
Hong and Han [31]	2016	Four amino acid-sensitive fingermark enhancement reagents (ninhydrin, 5-MTN, DFO, 1,2-IND)	Observed that NIN-ALA and MTN-ALA complexes induced colour changes to enhance fingermarks on fired cartridge casings, but photoluminescence was not observed. DFO-ALA and IND-ALA did not show any enhancement of fingermark.	Eccrine fingermark	0.38 caliber
Xu et al. [20]	2017	Electrostatic apparatus	The electrostatic adsorption method was successfully applied for latent fingermark visualisation on DAP-92 brass cartridge cases.	Natural fingermark	Unfired cartridge cases
Morrissey et al. [32]	2017	CA + BY40, CA + GB, or GB as a single process	Latent fingermark enhancement was possible using all three techniques.	Eccrine fingermark	Glock 19 semi-automatic pistol
Dove [33]	2018	GB electrodeposition	The GB electrodeposition is better at recovering identifiable latent fingermarks from fired cartridge cases than PD.	Natural fingermark	Fired cartridge case
Girelli et al. [7]	2018	CA + GB + BY40, CA + GB + Ardrox, deposition of PB films, and coating by aqueous electrolytes containing PP and SB	The sequence CA + GB + fluorescent dye (BY40 or Ardrox) is the best option to develop latent fingermarks on fired and unfired cartridge cases. PB and PP-SB did not produce satisfactory results for natural and eccrine-rich marks on brass surfaces.	Sebaceous and eccrine fingermarks	9 × 19 mm, model NTA, and a Glock 17 pistol
Bleay et al. [34]	2019	Disulphur dinitride, CA, GB, VMD, powder suspension	Reported that disulfur dinitride is an effective process for visualisation of fingermark (up to three months old), especially for those exposed to harsh environmental conditions. CA, GB, and VMD can also develop latent fingermarks from metal surfaces.	Natural fingermark	Bronze, brass, copper, and stainless steel
Christofidis et al. [18]	2019	VMD	VMD technique developed identifiable fingermarks on brass metal disks aged from a few days up to over a month old.	Natural fingermark	0.38 Smith and Wesson revolver/casing and brass metal plates
Brewer [35]	2019	VMD	VMD technique using silver metal ions produced the most identifiable fingermarks. However, the quantity of identifiable fingermarks was not significant.	Natural and sebaceous fingermark	Handgun
Pollitt et al. [36]	2020	Gold/Zinc and Silver/Zinc VMD	VMD has great potential in the recovery of aged fingermarks. Both Gold/Zinc and Silver/Zinc VMD treatments are effective methods of developing good contrast fingermarks on a ballistic brass surface.	Natural fingermark	9 mm Luger/Ballistic brass tiles
Costa et al. [19]	2020	Electrodeposition of PEDOT	Allowed the enhancement of visual contrast between the surface and the fingermark residues, including sebaceous, eccrine, and aged ones. Provided images of the developed fingermarks with high definition.	Sebaceous and eccrine fingermark	7.62 × 51 mm NATO
Wilkinson et al. [37]	2020	RLFT	RLFT presents promising prospects in fingermark science. The technique was capable of developing 17 of 147 (12%) identifiable fingermarks from fired cartridge cases.	Ungroomed fingermark	Fired ammunition and detonatedimprovised explosive devices
Pontone et al. [38]	2021	RLFT	Exactly 6 (3%) spent casings presented with identifiable fingermarks. About 112/195 (57%) of the spent casings presented with friction ridges although not fit for identification after RLFT processing.	Natural and sebaceous fingermark	Brass cartridge cases
Lam et al. [39]	2022	Disulfur dinitride, CA/BY40, and/or VMD	Overall, 121 out of 1 304 (9.3%) of natural fingermarks deposited were deemed identifiable post-firing and processing with RLFT.	Natural fingermark	Brass.223 Remington American Eagle
Exall et al. [22]	2022	RLFT, CA + BY40	CA + BY40 developed better identifiable fingermarks than the RLFT.	Natural fingermark	9 × 19 mm and 0.45 cal; 5.56 × 45 mm cartridge cases; 7.62 × 51 mm calibre cartridge cases
Wong et al. [40]	2023	RLFT	The quality of fingermarks developed post-RLFT application is improved with the elapsed time. Posited that more fingermarks (*n* = 35, 29.2%) increased in quality 7 days after RLFT enhancement compared to the fingermarks (*n* = 22, 18.3%) that increased in quality 2 days after enhancement.	Natural fingermark	Not specified
Caven et al. [41]	2023	RLFT	RLFT developed natural latent fingermark better on brass than steel metal plate. RLFT performs better at developing natural fingermarks on brass and steel plates than eccrine-based fingermarks.	Natural, eccrine, and sebaceous fingermark	Brass and steel metal plates

GB: gun bluing; AHP: acidified hydrogen peroxide; CA: cyanoacrylate fuming; BY40: basic yellow 40; PD: palladium deposition; RUVIS: reflected ultraviolet imaging system; UV: ultraviolet; CPF: cold patination fluid; 5-MTN: 5-methylthioninhydrin; DFO: 1,8-diazafluoren-9-one; 1,2-IND: 1,2-indanedione; PB: Prussian blue; PP: potassium permanganate; SB: sodium bicarbonate; VMD: vacuum metal deposition; PEDOT: poly(3,4-ethylene dioxythiophene); RLFT: RECOVER Latent Fingerprint Technology; NIN-ALA: ninhydrin-*L*-alanine; NTA: non-toxic ammunition; NATO: North Atlantic Treaty Organization.

Despite the difficulties that the surfaces of fired and unfired cartridge cases present to fingermark examiners, some studies [7, 14, 15] have been successful at developing latent fingermarks from these casings using different standalone and combined or sequential fingermark enhancement techniques. The efficiency of six different fingermark enhancement techniques (CA + BY40; CA + GB + BY40; GB alone; CA + PD; PD only; and Powder suspension) was investigated by Dominick and Laing [14] to understand their ability to develop latent fingermarks from unfired cartridges. They reported that the technique suitable for the enhancement of latent fingermarks from unfired cartridge cases was the sequential application of either: CA followed by GB and BY40, or CA followed by PD. Similarly, Girelli et al. [15] affirmed that the CA technique followed by the GB and the application of BY40 was efficient at developing latent fingermarks from both fired and unfired cartridge cases which was consistent with a later study published in 2018 [7]. Preliminarily, Morrissey et al. [32] developed latent fingermarks from fired and unfired casings (9 mm caliber) using three different techniques: CA + BY40, CA + GB, and GB alone. Although all three techniques produced quality results, Morrissey et al. [32] alluded that the GB (conc. 50% v/v) technique singly outperforms the sequential application of CA + BY40 or CA + GB. This finding was in agreement with the comparative study conducted by Bhaloo et al. [24] where the authors reported that GB alone or PD independently performs more efficiently than the CA followed by the BY40 technique. GB solution comprises three core ingredients notably an acid (phosphoric acid and sulfamic acid), a cupric salt, and a selenious acid which collectively form an oxidizing agent with the capability of reacting with metal surfaces to form a copper selenide coating. Being a rust-induced reaction (H_2_SeO_3_ + 4H^+^ + 4e^−^ → Se + 3H_2_O) [32], GB like AHP is sensitive and effective at developing eccrine latent fingermarks than sebaceous fingermarks since the oils present in the latter inhibit the oxidation reaction.

Amata et al. [30] used the CA technique alone to develop and visualise latent fingermark on the trigger of a firearm (Mauser Werke 90 DA/9 mm Parabellum) in a real-life case scenario. The CA process was conducted in a Foster and Freeman fuming chamber (MVC—3000) using a Cyano Blum liquid glue at 80%. Paine et al. [25] experimented and reported that a relative humidity of ⁓80% is sufficient to catalyse the visualisation of latent fingermarks on shell casings and other non-porous substrates. Following the CA process, Amata et al. [30] obtained an identifiable fingermark from the trigger of a firearm. The visibility of the developed fingermarks was further enhanced with an ALS particularly white light and ultraviolet (UV) light before digital photography. The findings by Amata et al. [30] is contrary to the study conducted by Leintz and Bond [29] to visualise fingermarks from fired cartridge cases using a reflected ultraviolet imaging system (RUVIS) with a wavelength of 254 nm. It was reported that the RUVIS which uses shortwave UV light was unsuccessful at developing fingermarks from fired brass cartridge cases. Comparative to the performance of RUVIS, it was shown that optical interference and digital colour mapping aided by natural daylight were successful at enhancing fingermarks from only 3/12 fired brass cartridge cases that is similar to the findings reported by Bond [26].

Dove [33] added that the electric treatment of cartridge cases prior to the sequential application of CA and GB performs better at developing clearer continuous ridges than just the sequential application of CA and GB. Specifically, Dove established that the electrodeposition of a GB on a fired cartridge case improves the efficiency of the subsequent fingermark enhancement techniques (CA followed by BY40) compared to the electrodeposition of palladium on the casings. Consistently, Bhaloo et al. [24] affirmed that the electrodeposition of palladium supersedes the conventional sequential use of CA followed by BY40. It can be hypothesised that the adoption of the sequence: electrodeposition of GB or palladium followed by the application of CA + BY40 can improve the development and visualisation of quality fingermarks from fired and unfired cartridge cases. Costa et al. [19] electrochemically deposited only poly(3,4-ethylene dioxythiophene) at low potential (0.9 V at 180 s) on the surfaces of fired brass cartridge cases to advance the enhancement of latent fingermark. Despite the destructive nature of this electrochemical deposition approach, its potential in forensic fingermark enhancement is worth exploring because it is uncomplicated and requires less time to yield good-quality fingermarks on fired brass cartridge cases.

Different amino acid-sensitive fingermark enhancement reagents notably 1,2-indanedione-*L*-alanine (IND-ALA), ninhydrin-*L*-alanine (NIN-ALA), 1,8-diazafluoren-9-one-*L*-alanine (DFO-ALA), and 5-methylthioninhydrin-*L*-alanine (MTN-ALA) were investigated to ascertain their ability to visualise eccrine latent fingermark on fired cartridge cases [31]. The foregoing reagents were prepared and sprayed on fired cartridge cases bearing eccrine fingermarks. The visualisation of the latent fingermarks was chiefly influenced by the reagents' sensitivity and ability to detect trace metal ions on the surface of the brass ammunition. Following the experimentation, Hong and Han [31] identified that MTN-ALA performs better than NIN-ALA with regards to the enhancement and visualisation of quality latent fingermarks on spent casings which accords with the works of Almog et al. [42] whose investigation was rather performed on paper instead of a brass metal surface. Particularly, an estimated 30% (*n* = 31) of fired cartridge cases (*n* = 102) that were treated with MTN-ALA presented with clear continuous fingermark ridges that can permit identification whereas only ⁓7% (*n* = 7) of fired cartridge cases (*n* = 101) treated with NIN-ALA produced identifiable fingermark ridges [31]. On the contrary, IND-ALA and DFO-ALA were found to be insensitive to trace metal ions and therefore could not develop and visualise fingermarks on spent cartridges cases [31]. This drawback can be linked to the fact that the catalyst (cellulose) [43] for the trace metallic ion reaction with DFO-ALA and IND-ALA is deficient in brass cartridge cases. Even on paper where the presence of cellulose is not uncommon, the quality of fingermarks developed with IND-ALA and DFO-ALA was noted to be very minimal which corresponds with the results that were disclosed by an earlier study [44].

Further, myriad standalone fingermark enhancement techniques have also been explored to ascertain their potential to visualise latent fingermarks on cartridge cases (fired and unfired). A preliminary study was conducted by Christofidis et al. [18] to evaluate the potential of VMD in the enhancement of latent fingermarks on ballistic materials. A combination of gold/zinc and silver/zinc metals were deposited in series on the brass cartridge cases bearing the natural latent fingermark. Following the deposition of the foregoing metal ions, Christofidis et al. [18] successfully demonstrated the effectiveness of the VMD technique in enhancing fingermarks with clear continuous ridges. The potential and reliability of the VMD technique were further explored by Pollitt et al. [36]. In support of the findings reported by the previous researchers [18], the works of Pollitt et al. [36] affirmed that the VMD technique using gold/zinc and silver/zinc metals was not only sensitive but efficient at enhancing 2-month-aged natural latent fingermarks from fired and unfired cartridge cases. Brewer [35] also compared four different metal ions (gold-zinc, silver, sterling silver, and copper-zinc) for the VMD technique and investigated their efficiency in developing latent fingermarks from fired cartridge cases. The authors reported that ⁓12% and 28% of the total number of fired cartridge cases (*n* = 200) presented with good and moderate quality fingermarks, respectively, with the most successful results obtained with the silver metal ions. The consistency in the findings from the scholars [18, 34–36] shows that the nondestructive VMD technique is reliable, rapid (process time of ˂10 min), and presents a great prospect for fingermark enhancement from cartridge cases.

An electrostatic method (using an electrostatic detection apparatus) which is originally used for the visualisation of footwear marks was optimised by Xu et al. [20] to develop aged (1, 4, and 10 days) natural latent fingermarks from unfired cartridge cases. To critically assess the performance of the modified electrostatic method (potential of +25 kW), Xu et al. [20] developed latent fingermark using two additional methods: the powder brushing method (with a light gentle brushing action) and treatment with 2% (w/v) silver nitrate-ethanol solution by dipping for ⁓2–5 s. The cartridge cases that were treated with the silver nitrate-ethanol solution were further cleaned with distilled water to remove excess chemical reagents and allowed to air dry. The results of the study analysis reflect that the enhancement efficiency of the powder method, the silver nitrate-ethanol solution method, and the electrostatic method vis-à-vis the enhancement of natural latent fingermarks aged 1 and 4 days was comparable. However, regarding natural fingermarks aged 10 days, Xu et al. [20] showed that the electrostatic detection method developed satisfactory identifiable fingermarks from the unfired cartridge cases whereas the remaining two methods (powder method and the silver nitrate-ethanol solution) produced no identifiable results. Although the modified electrostatic method described by Xu et al. [20] has proved efficient in enhancing latent fingermarks from unfired cartridge cases, its ability to develop latent fingermarks from fired cartridge cases was unsuccessful [24]. That notwithstanding, the electrostatic method exhibits promising potential in fingermark science and requires more research to ascertain its reliability in the enhancement of fingermarks from fired cartridge cases. The electrostatic method described by Xu et al. [20] and Bhaloo et al. [24] is advantageous to the electrolysis approach to fingermark enhancement tested by Nizam et al. [27]. The electrolysis reaction occurring in an acidic electrolyte solution destroys tool marks such as chamber marks and breech face marks on the fired cartridge cases which further preclude other relevant ballistics investigations such as firearm-ammunition linkage and shooter identification. A related study by Bond and Heidel [13] involved heating the fired cartridge case to 700°C coupled with a 2.5 kV potential application followed by a subsequent introduction of a conducting carbon powder. The above-described technique developed latent fingermarks from the fired cartridge case despite the time elapsed since the commission of the crime. The heating phase aids in inducing corrosion on the surface of the cartridge case due to the reaction between the electrolytes (ionic salts) in the fingermark’s residue and the brass surface. This induced corrosion somewhat contributes to successfully enhancing and visualising latent fingermarks from fired cartridge cases. For example, Bond [26] successfully enhanced latent fingermarks from fired cartridge cases with a digital colour mapping after the surfaces of the cartridge cases were preheated to a temperature of up to 600°C (optimal temperature was recorded as 250°C).

A rapid, simple, and inexpensive technique called CPF was proposed by James and Altamimi [21] to develop latent fingermarks on fired cartridge cases and unfired ammunition. Exactly 3% solution was prepared with distilled water from the CPF (mixture of selenium dioxide and nitric acid) procured from Priory Polishes in the UK. The cartridge cases were dipped into the patination fluid for ⁓90 s for latent fingermark enhancement. The findings reported by James and Altamimi [21] underscore that the latent fingermarks developed from the unfired ammunition were much clearer with continuous friction ridges relative to fingermarks developed from the fired cartridge cases. The authors further indicated that the enhancement of sebaceous fingermarks was comparatively better than the enhancement of eccrine fingermarks on both fired cartridge cases and unfired cartridges. Eccrine fingermark constituents present a higher tendency to evaporate with the elapse of time compared to the sebaceous fingermark. This could explain why developed sebaceous fingermarks are clearer than their eccrine counterparts. Comparatively, the CPF method although destructive works better than the traditional CA technique [21]. This assertion was made because latent fingermarks developed with CPF are more resistant to alterations and damage unlike those developed with the CA technique, which are more susceptible to damage post-enhancement.

In recent times, the potential of the RLFT produced by Foster and Freeman^®^ has also been researched for the enhancement of latent fingermark from metal surfaces and brass cartridge cases exposed to extreme heating [22, 37–39, 41]. The RLFT uses vapors of disulfur dinitride (S_2_N_2_) in a vacuum to develop latent fingermarks. A proof-of-concept study using the RLFT was conducted by Wilkinson et al. [37] to develop latent fingermarks on fired cartridge cases and detonated improvised explosive devices (IEDs). Out of the 147 fired casings, only 17 (12%) presented with good-state fingermarks following processing with the RLFT. This result was consistent with the findings reported by Pontone et al. [38]. Although the number of fired cartridge cases that presented with identifiable fingermarks was not substantial, Pontone et al. [38] mentioned that the RLFT would be a great addition to the numerous techniques adopted for the enhancement of latent fingermark on fired cartridge cases and metal surfaces. In a supporting study using the same technology but a larger pool of fingermarks (⁓1 540), Lam et al. [39] disclosed that only 102/652 (15.6%) fired brass cartridge cases contained identifiable fingermarks with continuous ridges post-treatment with the RLFT. The foregoing findings resonated with the results of Exall et al. [22] whose study preliminarily investigated the performance of the RLFT on the development of latent fingermarks from unfired ammunition and fired cartridge cases. Caven et al. [41] also evaluated the performance of the RLFT system in the development of latent fingermarks from metal plates to gain a baseline comprehension of its sensitivity and selectivity. The researchers alluded that the RLFT system performs better at recovering natural fingermarks (rather than only eccrine-based fingermarks) from brass metal plates than natural and eccrine-based fingermarks from steel metal plates. The efficiency of the RLFT was explored further by Wong et al. [40] to ascertain the effect of time elapsed on the quality and quantity of fingermarks developed. The authors enumerated that the quality of fingermarks developed post-RLFT application was improved with the elapsed time. For instance, the scholars [40] posited that more fingermarks (*n* = 35, 29.2%) increased in quality 7 days after RLFT development compared to the fingermarks (*n* = 22, 18.3%) that increased in quality 2 days after development. Following using RLFT for fingermark enhancement, the reagent (S_2_N_2_) may continue to interact with the latent fingermark residues over time leading to enhanced contrast and ridge detail visibility. Unsurprisingly, the effect of time since fingermark deposition on the cartridge case prior to the firing process negatively impacted the quality of the fingermark developed. For example, Wong et al. [40] reported that only 15 (12.5%) and 4 (3.3%) of fingermarks deposited 24 h and 1 h before firing, respectively, were suitable for identification. Interestingly, this finding contradicts the expectation that fingermarks deposited just 1 h before firing would exhibit better quality than those deposited 24 h earlier. The disulfur dinitride (S_2_N_2_) method also produced good-quality fingermarks on metal surfaces such as bronze, brass, copper, and stainless steel other than fired cartridge cases and unfired cartridges. Whereas the RLFT has a promising prospect as a latent fingermark developer, its current performance is unfortunately subpar to the best practice sequential fingermark enhancement technique (CA + GB + BY40) recommended for fingermark development from ballistic materials. Consequently, the optimisation of the parameters and functionality of the RLFT is necessary to improve its performance for the enhancement of latent fingermark on challenging metal surfaces including fired and unfired cartridge cases.

Current understanding of the review of results from existing literature demonstrates the challenges fingermark examiners and scholars encounter during the processing of fired and unfired cartridge cases for fingermark evidence. The most persistent reason accounting for this challenge is the process the cartridge cases go through during firearm discharge. For example, Bentsen et al. [16] and Girelli et al. [7], argued that factors such as the frictional contact between the cartridge and the walls of the chamber, ammunition-ammunition interaction inside the magazine of a firearm, blowback of hot gases (⁓3 000°C), and the physical impact of gunshot residues against the cartridge cases [45] contributes to the deterioration of fingermark on a fired cartridge case. The blowback of the hot gases alone is considered to be a major cause of fingermark destruction on spent cartridge cases. For instance, Girelli et al. [7] found that fingermarks recovered from cartridge cases that were manually passed through the firearm without undergoing the firing process presented excellent contrast and ridge clarity compared to the fingermarks developed from fired cartridge cases. This observation affirms that the frictional contact between the cartridge case and the walls of the firearm chamber and other parts of the firearm rarely interferes with the quality of the fingermarks compared to the blowback of hot gases. The hot gases are generated following the burning of propellant charge inside the cartridge cases. It is worth accenting that the intensity of the hot gases generated during the firing process is primarily influenced by the amount of propellant charge inside the cartridge case. As a result, cartridge cases with a higher quantity of propellant charge cause greater damage to the latent fingermark than those with a lower amount of propellant charge. Despite the odds, the fingermark evidence on these cartridge cases cannot be undervalued since it could be the only forensic proof to link a culprit to a crime scene or the victim.

## Limitations and recommendations for future directions

Studies that specifically investigate the impact of the firing process on the persistence of latent fingermark on spent casings are limited. Extensive research in this regard is therefore needed to inform the selection of suitable fingermark enhancement techniques to facilitate fingermark development from these problematic cartridge cases. The majority of success acclaimed in existing literature vis-à-vis fingermark enhancement from fired cartridge cases were performed under laboratory conditions that to a greater extent favour fingermark development. Although these experimentations are valid as proof-of-concept, the efficiency of the successful fingermark enhancement technique should be explored on real-life case samples to help understand and validate the full performance of these techniques.

Although both conventional and advanced fingermark enhancement techniques have been explored for the recovery of latent fingermarks from fired and unfired cartridges cases, we observed that the use of an ALS such as HandScope LED, CrimeScope, and Light amplification by stimulated emission of radiation (LASER), and other forensic light sources to further improve fingermark visualisation was deficient. The use of ALS as a complementary technique has been found to improve visualisation of enhanced fingermark [17, 30]. In addition to its core ability to improve the visibility of enhanced fingermark evidence, ALS is versatile (includes multiple wavelengths), reliable, portable, easy to use, and non-destructive. We, therefore, suggest that future research and operational forensic laboratories regularise using ALS as a complementary technique to increase the visibility of enhanced fingermark evidence.

Despite the development of current methods and the modification of existing techniques, we recommend that fingermark examiners prioritise adopting the sequential application of CA + GB + BY40 coupled with an appropriate ALS for the enhancement and visualisation of latent fingermarks from fired cartridge cases. This is imperative because it has a greater fingermark enhancement rate, it is cheap, quick, well-researched, and requires no special expertise.

## Conclusion

The development of latent fingermarks from fired and unfired cartridge cases plays a pivotal role in solving crimes, linking firearms to criminal activities, providing a lead in criminal cases, and contributing to safe justice delivery. As per data from our review, the most well-established method for enhancing latent fingermarks from fired and unfired cartridge cases is the sequential application of CA, followed by GB, then BY40. Over the last several years, more sophisticated and reassuringly reliable methods (such as RLFT, VMD, CPF, and PD) have emerged; these methods have proven successful in developing latent fingermarks from fired and unfired cartridge cases. We also observed that the electrostatic method of fingermark development presented contrasting results, necessitating extensive research. This review discussed the current scope of research, highlighted the limitations, and provided practical recommendations for future perspectives.
